# Chronic Ammonia Stress in Chinese Perch (*Siniperca chuatsi*): Oxidative Response, Nitrogen Metabolism, and Multi-Enzyme-Mediated Molecular Detoxification Defense Mechanisms

**DOI:** 10.3390/antiox14070768

**Published:** 2025-06-22

**Authors:** Yan Li, Ru Yang, Minghui He, Jianmei Su, Liwei Liu

**Affiliations:** 1 College of Fisheries, Chinese Perch Research Center, Engineering Research Center of Green Development for Conventional Aquatic Biological Industry in the Yangtze River Economic Belt, Ministry of Education, Huazhong Agricultural University, Wuhan 430070, China; 2023308110023@webmail.hzau.edu.cn (Y.L.); 15812958078@163.com (R.Y.); 2 Hubei Key Laboratory of Regional Development and Environmental Response, Faculty of Resources and Environmental Science, Hubei University, Wuhan 430062, China; 18380305308@163.com

**Keywords:** Chinese perch, ammonia nitrogen stress, oxidative stress, antioxidant enzymes, safe concentration, gene expression

## Abstract

Chinese perch (*Siniperca chuatsi*), an economically important freshwater fish in China, faces ammonia nitrogen stress under high-density aquaculture. This study investigated chronic ammonia nitrogen exposure effects on juvenile fish (95 ± 5 g) to establish safe concentration. Acute toxicity tests revealed a 96 h-LC_50_ of 12.91 mg/L ammonia nitrogen, with a safe concentration of 1.29 mg/L ammonia nitrogen (non-ionic ammonia: 0.097 mg/L). In 28-day chronic experiments with ammonia nitrogen levels at 0, 0.61, 1.29, and 2.58 mg/L, ammonia nitrogen induced hepatic oxidative stress, with total superoxide dismutase, catalase, and glutathione peroxidase activities and malondialdehyde content increasing proportionally to ammonia nitrogen concentration initially but declining over time. Concurrently, gill Na^+^-K^+^-ATPase activity was significantly suppressed, while the gene expression of ammonia transporters (*rhag*, *rhbg*, and *rhcg*) exhibited ammonia nitrogen concentration-dependent upregulation, inversely correlated with the exposure duration. Histological gill damage intensified at higher concentrations. Hepatic ammonia detoxification enzymes activities (asparagine synthase, glutamine synthetase, and glutamate dehydrogenase) and glutamine accumulation increased with ammonia nitrogen levels, aligning with gene expression trends, though enzyme activity diminished over time. Serum alanine aminotransferase and aspartate aminotransferase activities and their gene expressions rose with ammonia nitrogen levels, while total protein declined. These findings demonstrate that chronic ammonia nitrogen stress disrupts antioxidant capacity, osmoregulation, and nitrogen metabolism, compelling Chinese perch to mitigate toxicity via glutamine synthesis. To ensure sustainable aquaculture, ammonia nitrogen levels should remain below 1.29 mg/L under adequate dissolved oxygen conditions.

## 1. Introduction

Amino acids serve as fundamental building blocks in physiological processes, playing pivotal roles in protein synthesis, energy metabolism, and nitrogen homeostasis in teleost fishes [1,2]. While dietary proteins are hydrolyzed into amino acids to sustain vital functions, their catabolism inevitably generates ammonia as a toxic byproduct through deamination pathways [3,4]. Despite considerable advances in elucidating ammonia excretion mechanisms in teleosts, critical knowledge gaps persist regarding the molecular regulation of ammonia genesis in carnivorous species, particularly under intensive aquaculture conditions.

Chinese perch (*Siniperca chuatsi*) is a highly valued freshwater fish species in China [5]. It represents an ideal model for investigating ammonia nitrogen dynamics due to its high dietary protein requirements (45–55% crude protein content) and consequent elevated ammonia production [6,7]. Current industrial practices emphasizing high-density cultivation exacerbate environmental ammonia nitrogen fluctuations, with total ammonia nitrogen (TAN: NH_3_ + NH_4_^+^) concentrations frequently exceeding natural water levels (0–0.2 mg/L) [8] due to the cumulative effects of metabolic waste, feed residues, and microbial mineralization. The NH_3_/NH_4_^+^ equilibrium (NH_4_^+^ + OH^−^ ⇌ NH_3_·H_2_O ⇌ NH_3_ + H^+^), governed by pH and temperature [9,10], means that creates a chemical milieu where even minor environmental perturbations can trigger toxicological cascades.

Ammonia nitrogen toxicity manifests through multiple pathological pathways: (1) oxidative stress via reactive oxygen species (ROS) overproduction and compromised antioxidant defenses [11,12]; (2) immunosuppression characterized by intestinal inflammation and dysregulated cytokine expression [13]; and (3) systemic dysfunction evidenced by hematological abnormalities [14], histopathological lesions [15], and physiological impairment [16]. Existing research on Chinese perch predominantly focuses on acute toxicity thresholds, while chronic exposure effects under realistic aquaculture scenarios remain poorly characterized [8,17]. This knowledge gap hinders the development of science-based water quality management protocols.

This study employs static aquatic toxicity testing to establish the 96 h-LC_50_ and safe concentration (SC) of ammonia nitrogen for juvenile Chinese perch. Through systematic evaluation of chronic ammonia nitrogen exposure impacts, we aim to decipher the physiological adaptation mechanisms in this commercially vital species and provide actionable thresholds for optimizing intensive rearing systems. Our findings advance the theoretical framework of ammoniotelic regulation in carnivorous teleosts while offering practical solutions for sustainable aquaculture.

## 2. Materials and Methods

### 2.1. Experimental Animals and Management

Chinese perch (initial body weight 95 ± 5 g) was obtained from Baishazhou aquatic product market (Wuhan, China). The specimens were acclimated in 1.8 m-diameter × 1 m-height circular fiberglass tanks with recirculating water systems, maintaining water temperature at 18.0 ± 0.5 °C and dissolved oxygen at 7.0 ± 0.3 mg/L. Commercial feed (crude protein ≥ 48%) was administered daily at 09:00 (1% of total biomass). During the 14-day acclimation period, 50% water exchange was performed daily at 12:00.

### 2.2. Acute Toxicity Test (96 h-LC_50_ Determination)

Chinese perch, with an average weight of 95 ± 5 g, was acclimatized to a temperature of 25 °C. A stock solution of NH_4_Cl at a concentration of 10 g/L was prepared and further diluted to achieve the desired concentrations. The initial ammonia nitrogen levels were monitored before the commencement of the experiment to ensure that the water ammonia nitrogen content remained below 0.01 mg/L. Four different concentrations of ammonium chloride (40, 45, 50, and 60 mg/L) as well as a control group (0 mg/L) were established, corresponding to measured total ammonia nitrogen concentrations of 10.39, 11.84, 13.46, 15.03 and 0 mg/L. The pH concentration ranged from 7.86 to 8.14 during the experiment. Each concentration level consisted of three replicates, and each replicate contained 10 Chinese perch larvae. The experimental water depth was 0.8 m. Prior to the experiment, feeding was withheld for 24 h. The ammonia nitrogen levels in the experimental tanks were monitored every 6 h by sampling the water, diluting it tenfold, and analyzing the ammonia nitrogen concentration using spectrophotometry (ND-1000, Thermo Fisher Scientific, Waltham, MA, USA). Any deceased individuals were promptly removed during the experiment, and the mortality rate of young Chinese perch after 96 h was recorded. Statistical analysis was performed using SPSS 25.0 software, employing linear interpolation to determine the 96 h-LC_50_ value and estimating the safe concentration (SC) of ammonia nitrogen for the safety of the Chinese perch, calculated as SC = 0.1 × 96 h-LC_50_.

### 2.3. Chronic Exposure Experimental Design

For the chronic exposure, three distinct levels of ammonia nitrogen concentration were established: a low ammonia nitrogen group with 0.5 SC (0.61 mg/L), a medium ammonia nitrogen group with 1 SC (1.29 mg/L), and a high ammonia nitrogen group with 2 SC (2.58 mg/L), alongside a control group (0 mg/L) devoid of added ammonia nitrogen. Each group consisted of three replicates. Healthy juvenile Chinese perch specimens of similar weight were selected and placed in 12 breeding tanks (each with a volume of 0.5 m^3^) containing 20 individuals per barrel. At 09:00 am, the fish were fed an amount equivalent to 1% of their body weight. Subsequently, the water in the tanks was completely replaced, and the appropriate concentration of ammonia nitrogen solution was reintroduced to maintain consistency with the experimental design. The pH value of each experimental group was between 8.08 and 8.26 during the experimental period. Water samples (50 mL) were collected from the middle of each experimental tank before feeding and after water replacement to measure the ammonia nitrogen levels. After 15 and 30 days of exposure, a subset of fish from each tank were subjected to a 24 h fasting period, followed by anesthesia using MS-222 (150 mg/L) and subsequent body weight measurement. Six fish were randomly selected from each group for blood sampling, which was stored overnight at 4 °C. Serum samples were obtained by centrifugation at 3000× *g* for 20 min. Liver tissues were rapidly frozen in liquid nitrogen for subsequent gene expression and enzyme activity analyses and stored at −80 °C.

All experiments were conducted in accordance with the Guidelines for the Care and Use of Laboratory Animals in China. All experimental protocols followed local animal welfare regulations and institutional guidelines.

### 2.4. Physiological and Biochemical Assays

Various biochemical parameters, including total protein (TP) content, Na^+^-K^+^-ATPase activity, catalase (CAT) activity, total superoxide dismutase (T-SOD) activity, glutathione peroxidase (GSH-Px) activity, acid phosphatase (ACP) activity and malondialdehyde (MDA) content, were assessed using commercial kits from the Nanjing Jiancheng Bioengineering Research Institute (Nanjing, China). The Coomassie brilliant blue method was employed for the TP content measurement, while CAT activity and MDA content were quantified using the ammonium molybdate and thiobarbituric acid methods, respectively. T-SOD activity was determined using the hydroxylamine method, and GSH-Px activity was measured using a colorimetric assay. ACP activity was assessed using a microplate method. Asparagine synthase (AS) activity was analyzed using a kit from Shanghai Qiyi Biological Technology Co., Ltd. (Shanghai, China), while glutamate dehydrogenase (GDH) activity and glutamine (Gln) content were measured using kits from Hefei Laier Biological Technology Co., Ltd. (Hefei, China). Glutamine synthetase (GS) activity was assessed using a kit from Beijing Solarbio Science & Technology Co., Ltd. (Beijing, China). Alanine aminotransferase (ALT) and aspartate aminotransferase (AST) activities were determined using an automatic biochemical analyzer (BioDot, Irvine, CA, USA) with kits from Zhongsheng Beikong Biotechnology Co., Ltd. (Beijing, China).

### 2.5. Gene Quantification Analysis

#### 2.5.1. RNA Extraction

Tissue samples (30–50 mg) were weighed into 2 mL EP tubes, followed by rapid addition of 1 mL RNAiso Plus and two glass beads. The samples were immediately homogenized using a pre-cooled tissue homogenizer. After homogenization, the lysates were incubated at room temperature for 5 min and centrifuged at 12,000× *g* (4 °C, 5 min). The supernatant was carefully transferred to a new 1.5 mL EP tube. Chloroform (200 μL) was added to the lysate, vortexed until emulsified, and incubated at room temperature for 5 min. The mixture was centrifuged at 12,000× *g* (4 °C, 15 min), yielding three distinct layers: a colorless upper aqueous phase (RNA), a white interphase (protein/DNA), and a colored lower organic phase. The aqueous layer was transferred to a fresh tube, and an equal volume of isopropanol was added. After thorough mixing, the solution was incubated at room temperature for 10 min and centrifuged at 12,000× *g* (4 °C, 10 min). The supernatant was discarded, and the RNA pellet was washed with 75% ethanol, followed by centrifugation at 12,000× *g* (4 °C, 5 min). The pellet was air-dried and dissolved in RNase-free water.

#### 2.5.2. RNA Quality Assessment

The RNA concentration and purity were determined using a multifunctional microplate reader (SynergyHTX, Biodot, Irvine, CA, USA) by measuring absorbance ratios at 260 nm and 280 nm (about 1.8–2.2). For integrity analysis, 1.2% agarose gel was prepared with 1 × TAE buffer (diluted from 50 × TAE stock using ultrapure water). RNA samples (1 μL) were mixed with 3 μL of GelRed-containing loading buffer and electrophoresed with DL2000 DNA Marker (Biodragon, Beijing, China). Electrophoresis was performed at 220 V for 10 min. The gel was visualized under UV light using a gel imaging system (Quantum, Vilber, Paris, France) to assess RNA integrity based on the intact 28S, 18S, and 5S ribosomal RNA bands. Qualified RNA samples were aliquoted and stored at −80 °C for subsequent reverse transcription and gene expression analysis.

#### 2.5.3. cDNA Synthesis

Total RNA (1 μg) was reverse-transcribed into cDNA using a two-step reverse transcription kit from Vazyme Biotech Co., Ltd. (R223-01, Nanjing, China). Genomic DNA was removed by incubating the RNA with 4 × gDNA wiper Mix (4 μL in a 16 μL reaction system, 42 °C, 2 min). Subsequently, 5 × HiScript II qRT SuperMix II (4 μL) was added, and the reaction was performed at 50 °C for 15 min, followed by enzyme inactivation at 85 °C for 5 s. The quality of cDNA product was verified by 1.2% agarose gel electrophoresis (exhibited a single and bright band) and stored at −20 °C [10].

### 2.6. qRT-PCR Primer Design

In this study, the cDNA sequences of Chinese perch genes were analyzed to design corresponding qRT-PCR primers. The cDNA sequences were obtained from our laboratories’ Chinese perch gene pool, as reported by He et al. [18]. The primers for target genes were designed using Primer Perimer 5.0 software and then synthesized by Sangon Biological Engineering Technology and Services Co., Ltd. (Shanghai, China). The primer design criteria included specifications such as a primer length of 18–27 base pairs, an amplification length of 80–200 base pairs, avoidance of base A at the 3′ end of the primer, a GC content of 40–60% in the primer sequence, and ensuring that the difference in Tm values between the forward and reverse primers did not exceed 5 °C. The primer sequences for quantitative fluorescence of *Siniperca chuatsi* are detailed in Table 1. Relative gene expression data were normalized using the ribosomal protein L13A reference gene (*rpl13a*) and calculated via the 2^−ΔΔCT^ method

### 2.7. Statistical Analysis

The data are expressed as mean ± SEM. Statistical comparisons were performed using IBM SPSS statistics 25.0 with the following methods: Between-group differences: One-way ANOVA followed by Duncan’s multiple range test. Within-group temporal differences: Paired one-sample *t*-test. The significance thresholds were set at *p* < 0.05. Significant differences in the same ammonia nitrogen group at different times were marked with different capital letters, and significant differences among different ammonia nitrogen groups at the same time were marked with different lowercase letters.

## 3. Results

### 3.1. The Results of 96 h-LC_50_ Determination of Juvenile Chinese Perch

As shown in Table 2, juvenile Chinese perch exposed to varying concentrations of ammonia nitrogen over a 96 h period, with observed mortality rates of 0%, 20%, 60%, and 100%, respectively. The data indicated a positive correlation between elevated ammonia nitrogen levels and increased mortality rates. Through regression analysis, the 96 h median lethal concentration (96 h-LC_50_) of total ammonia nitrogen was determined to be 12.91 mg/L, while its safe concentration was established at 1.29 mg/L, which corresponds to 0.097 mg/L of non-ionic ammonia.

### 3.2. Different Ammonia Nitrogen Level Affect Chinese Perch Antioxidant Activity

**Figure 1:**
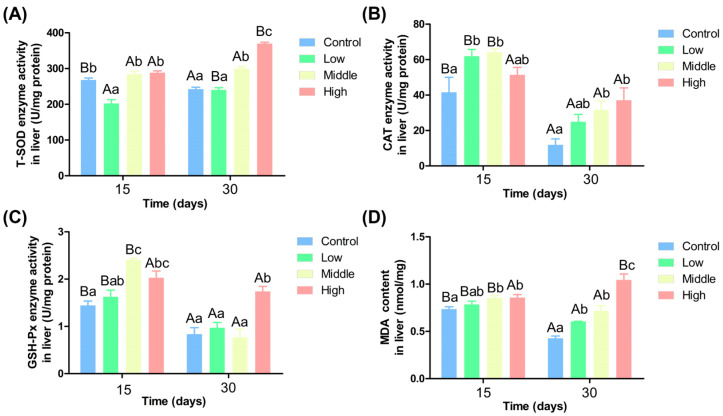
Activity of antioxidant enzymes in liver under different ammonia nitrogen conditions. (**A**) Enzyme activity of T-SOD in liver; (**B**) enzyme activity of CAT in liver; (**C**) enzyme activity of GSH-Px in liver; (**D**) the content of MDA in liver. Significant differences in the same ammonia nitrogen group at different times were marked with different capital letters (*p* < 0.05), and significant differences among different ammonia nitrogen groups at the same time were marked with different lowercase letters (*p* < 0.05). Those marked with the same letter indicate no significant difference between groups (*p* < 0.05).

Ammonia nitrogen affects liver function by regulating antioxidant enzymes. On the 15th day, T-SOD activity in the low ammonia nitrogen group was significantly lower than in the other groups (*p* < 0.05) (Figure 1A). On the 30th day, T-SOD activity in the middle and high ammonia nitrogen groups was significantly higher than in the control and low ammonia nitrogen groups and increased with ammonia nitrogen concentration (*p* < 0.05). While T-SOD activity decreased significantly over time in the control group, it increased significantly in the low and high ammonia nitrogen groups (*p* < 0.05). Figure 1B indicates that on the 15th day, CAT activity was significantly higher in the low and middle ammonia nitrogen groups than in the control group (*p* < 0.05). On the 30th day, CAT activity was significantly higher in the middle and high ammonia nitrogen groups than in the control group, showing a concentration-dependent increase (*p* < 0.05). CAT activity decreased significantly over time in the control, low, and middle ammonia nitrogen groups (*p* < 0.05).

Figure 1C shows that on the 15th day, GSH-Px activity was significantly higher in the middle and high ammonia nitrogen groups than in the control group (*p* < 0.05). On the 30th day, GSH-Px activity in the high ammonia nitrogen group was significantly higher than in all other groups (*p* < 0.05). GSH-Px activity decreased significantly over time in the control, low, and middle ammonia nitrogen groups (*p* < 0.05). Figure 1D shows that MDA content was significantly higher in the middle and high ammonia nitrogen groups than in the control group (*p* < 0.05) and increased with rising ammonia nitrogen concentration. On the 30th day, MDA content was significantly higherin the low, middle and high ammonia nitrogen groups than in the control group (*p* < 0.05). Over time, MDA content increased significantly in the high ammonia nitrogen group but decreased significantly in the other three groups (*p* < 0.05).

### 3.3. The Influence of Different Ammonia Nitrogen Levels on Chinese Perch Blood Biochemical

**Figure 2:**
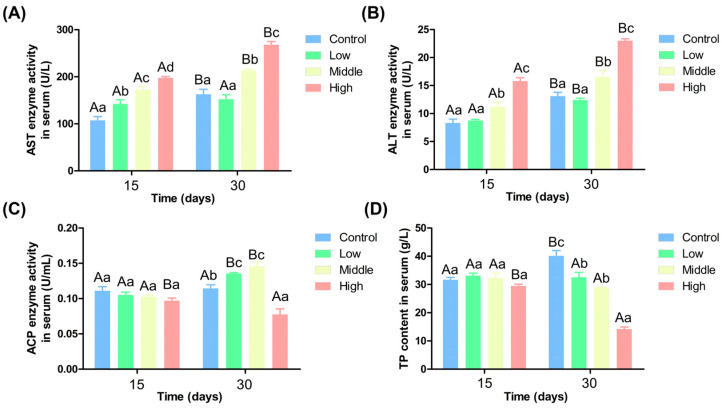
Activity of serum biochemical indexes of Chinese perch under different ammonia nitrogen conditions. (**A**) Enzyme activity of AST in serum; (**B**) enzyme activity of ALT in serum; (**C**) enzyme activity of ACP in liver; (**D**) the content of TP in serum. Significant differences in the same ammonia nitrogen group at different times were marked with different capital letters (*p* < 0.05), and significant differences among different ammonia nitrogen groups at the same time were marked with different lowercase letters (*p* < 0.05). Those marked with the same letter indicate no significant difference between groups (*p* < 0.05).

Figure 2A shows that the AST activity increased with ammonia nitrogen concentration on the 15th day, and there was a significant difference among them (*p* < 0.05). On the 30th day, AST activity was significantly higher in middle and high ammonia nitrogen groups than in the low ammonia nitrogen and control groups (*p* < 0.05). Except for the low ammonia nitrogen group, AST activity increased significantly over time in the other three groups (*p* < 0.05). Figure 2B shows that on the 15th day and 30th day, ALT activity was significantly higher in the middle and high ammonia nitrogen groups than in the control and low ammonia nitrogen groups (*p* < 0.05) and increased with ammonia nitrogen concentration. ALT activity increased significantly over time in all four groups with time (*p* < 0.05).

For ACP activity on the 15th day, it showed a decreasing trend with increasing ammonia nitrogen concentration, but differences among the groups were no significant (*p* > 0.05) (Figure 2C). On the 30th day, ACP activity in the low and middle ammonia nitrogen groups was significantly higher than in the control group, while its activity in the high ammonia nitrogen group was significantly lower than in the control group (*p* < 0.05). In addition to the high ammonia nitrogen group and control group, ACP activity increased significantly over time in the other two groups (*p* < 0.05). Figure 2D indicates no significant difference in TP content among groups on the 15th day (*p* > 0.05). On the 30th day, TP content decreased with increasing ammonia nitrogen concentration. TP content in the high ammonia nitrogen group was significantly lower than in the other three groups, and TP content in the low and middle ammonia nitrogen groups was also significantly lower than in the control group (*p* < 0.05). TP content decreased significantly over time in the high ammonia nitrogen group but increased significantly in the control group (*p* < 0.05).

### 3.4. Effects of Different Ammonia Nitrogen Levels on Liver Enzyme Activities of Chinese Perch

**Figure 3:**
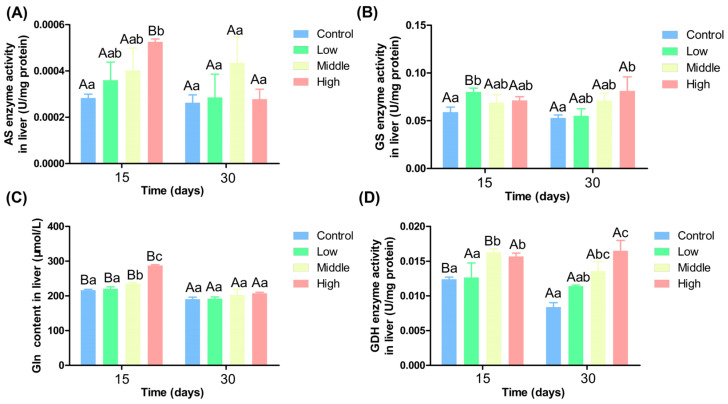
Liver transaminase activity under different ammonia nitrogen levels. (**A**) Enzyme activity of AS in liver; (**B**) enzyme activity of GS in liver; (**C**) the content of Gln in liver; (**D**) enzyme activity of GDH in liver. Significant differences in the same ammonia nitrogen group at different times were marked with different capital letters (*p* < 0.05), and significant differences among different ammonia nitrogen groups at the same time were marked with different lowercase letters (*p* < 0.05). Those marked with the same letter indicate no significant difference between groups (*p* < 0.05).

Figure 3A shows that on the 15th day, AS activity increased with ammonia nitrogen concentrations, and AS activity in the high ammonia nitrogen group was significantly higher than in the control group (*p* < 0.05). On the 30th day, no significant differences existed among groups (*p* > 0.05). AS activity decreased significantly over time only in the high ammonia nitrogen group (*p* < 0.05). Figure 3B indicates that on the 15th day, GS activity in the low ammonia nitrogen was significantly higher than in the control group (*p* < 0.05), while no significant differences existed among the other groups (*p* > 0.05). Over time, GS activity decreased significantly only in the low ammonia nitrogen group (*p* < 0.05), with no significant changes in the other groups (*p* > 0.05).

Figure 3C indicates that on the 15th day, Gln content was significantly higher in the middle and high ammonia nitrogen groups than in the control and low ammonia nitrogen groups (*p* < 0.05). On the 30th day, no significant differences in Gln content were observed among groups (*p* > 0.05). Gln content decreased significantly over time in all four groups (*p* < 0.05). As shown in Figure 3D, on the 15th day, GDH activity was significantly higher in the middle and high ammonia nitrogen groups than in the control and low ammonia nitrogen groups (*p* < 0.05). On the 30th day, GDH activity in the high ammonia nitrogen group was significantly higher than in the control and low ammonia nitrogen groups, and GDH activity in the middle ammonia nitrogen group was significantly higher than in the control group (*p* < 0.05). GDH activity decreased significantly over time in the control and middle ammonia nitrogen groups (*p* < 0.05).

### 3.5. Effects of Different Ammonia Nitrogen Levels on Gene Expression in the Liver of Chinese Perch

**Figure 4:**
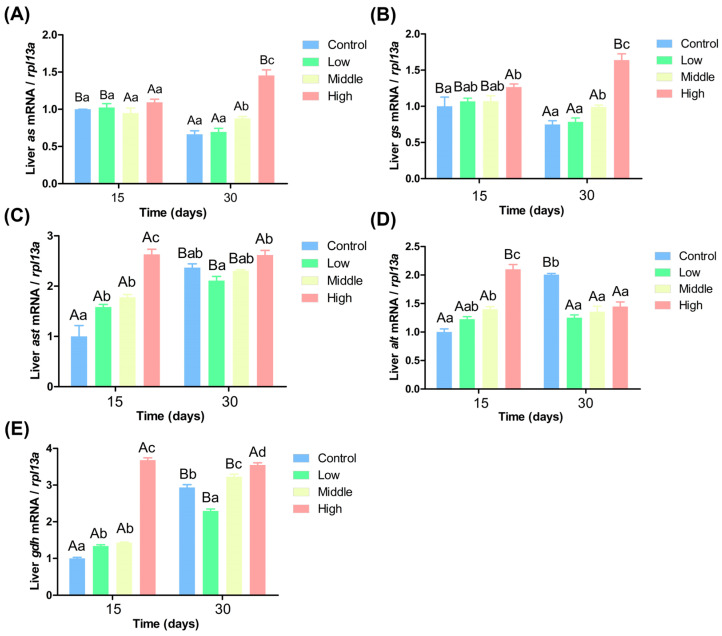
Liver gene expression under different ammonia nitrogen conditions. (**A**) Gene expression of *as* in liver; (**B**) gene expression of *gs* in liver; (**C**) gene expression of *ast* in liver; (**D**) gene expression of *alt* in liver; (**E**) gene expression of *gdh* in liver. Significant differences in the same ammonia nitrogen group at different times were marked with different capital letters (*p* < 0.05), and significant differences among different ammonia nitrogen groups at the same time were marked with different lowercase letters (*p* < 0.05). Those marked with the same letter indicate no significant difference between groups (*p* < 0.05).

Ammonia nitrogen significantly impacted the expression levels of genes related to nitrogen metabolism in the liver (Figure 4). Figure 4A shows that on the 15th day, *as* gene expression showed no significant differences among the four groups (*p* > 0.05). On the 30th day, *as* gene expression in the high ammonia nitrogen group was significantly higher than in the other three groups, and its expression in middle ammonia nitrogen group was significantly higher than in the control and low ammonia nitrogen groups (*p* < 0.05). Over time, *as* gene expression increased significantly in the high ammonia nitrogen group but decreased significantly in the control and low ammonia nitrogen groups (*p* < 0.05). Figure 4B indicates that on the 15th day, *gs* gene expression in the high ammonia nitrogen group was significantly higher than in the control group (*p* < 0.05), with no significant differences among the other groups (*p* > 0.05). On the 30th day, the high ammonia nitrogen group had the highest *gs* gene expression, followed by the middle ammonia nitrogen group, both of which had significantly higher expression than the control and low ammonia nitrogen groups (*p* < 0.05). Over time, *gs* gene expression significantly increased in the high ammonia nitrogen group but dramatically decreased in the other three groups (*p* < 0.05).

Figure 4C shows that on the 15th day, *ast* gene expression increased with ammonia nitrogen concentration. *ast* gene expression in the high ammonia nitrogen group was significantly than in the other three groups, and *ast* gene expression in the low and middle ammonia nitrogen groups was significantly higher than in the control group (*p* < 0.05). On the 30th day, *ast* gene expression in the high ammonia nitrogen group remained significantly higher than in the low ammonia nitrogen group (*p* < 0.05), but no significant differences were found among the other groups (*p* > 0.05). Over time, *ast* gene expression increased significantly in all groups except the high ammonia nitrogen group (*p* < 0.05). Figure 4D reveals that on the 15th day, *alt* gene expression increased with ammonia nitrogen concentration, *alt* gene expression in the high ammonia nitrogen group was significantly higher than in the other three groups, and expression in the middle ammonia nitrogen group was significantly higher than in the control group (*p* < 0.05). On the 30th day, *alt* gene expression in the control group was significantly higher than in the other three groups (*p* < 0.05), among which no significant differences existed (*p* > 0.05). Over time, *alt* gene expression increased significantly in the control group but decreased dramatically in the high ammonia nitrogen group (*p* < 0.05). Figure 4E indicates that *gdh* gene expression increased with ammonia nitrogen concentration, *gdh* gene expression in the high ammonia nitrogen group was significantly higher than in the control group. On the 30th day, significant differences existed among all groups: *gdh* gene expression in the low ammonia nitrogen group was significantly lower than in the control group, while expression in the medium and high ammonia nitrogen groups was significantly higher than in the control group (*p* < 0.05). Over time, *gdh* gene expression increased significantly in all groups except the high ammonia nitrogen group (*p* < 0.05).

### 3.6. Impact of Varying Ammonia Nitrogen Concentrations on the Activity and Expression of Gill Filase in Chinese Perch

**Figure 5:**
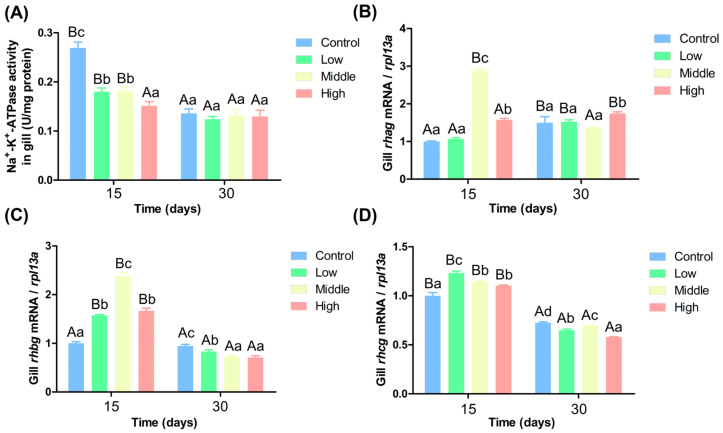
Gill enzyme activity and gene expression under different ammonia nitrogen levels. (**A**) Enzyme activity of Na^+^-K^+^-ATPase in gill; (**B**) gene expression of *rhag* in gill; (**C**) gene expression of *rhbg* in gill; (**D**) gene expression of *rhcg* in gill. Significant differences in the same ammonia nitrogen group at different times were marked with different capital letters (*p* < 0.05), and significant differences among different ammonia nitrogen groups at the same time were marked with different lowercase letters (*p* < 0.05). Those marked with the same letter indicate no significant difference between groups (*p* < 0.05).

Ammonia nitrogen significantly impacted gill filament Na^+^-K^+^-ATPase activity and the expression levels of genes related to ammonia transport (Figure 5). Figure 5A shows that on the 15th day, Na^+^-K^+^-ATPase activity declined with increasing ammonia nitrogen concentrations. Na^+^-K^+^-ATPase activity in the control group was significantly higher than in the other three groups, and its activity in the low and middle ammonia nitrogen groups were significantly higher than in the high ammonia nitrogen group (*p* < 0.05). On the 30th day, no significant differences in Na^+^-K^+^-ATPase activity existed among groups (*p* > 0.05). Over time, Na^+^-K^+^-ATPase activity decreased significantly in the control, low, and middle ammonia nitrogen groups (*p* < 0.05), while its activity in the high ammonia nitrogen group showed no significant change (*p* > 0.05). Figure 5B indicates that on the 15th day, *rhag* gene expression in the middle and high ammonia nitrogen groups was significantly higher than in the control group and low ammonia nitrogen groups (*p* < 0.05). On the 30th day, *rhag* gene expression in the high ammonia nitrogen group was significantly higher than in the other three groups. Over time, only *rhag* gene expression in the middle ammonia nitrogen group decreased significantly (*p* < 0.05).

Figure 5C shows that on the 15th day, *rhbg* gene expression in the middle ammonia nitrogen group was significantly higher than in the other three groups, and its expression in the low and high ammonia nitrogen groups was significantly higher than in the control group (*p* < 0.05). On the 30th day, *rhbg* gene expression in the middle and high ammonia nitrogen groups was significantly lower than in the other two groups, and its expression in the low ammonia nitrogen group was significantly lower expression than in the control group (*p* < 0.05). Over time, *rhbg* gene expression decreased significantly in the low, middle, and high ammonia nitrogen groups, while there was no significant change in the control group. (*p* < 0.05). Figure 5D indicates that on the 15th day, the expression levels of the *rhcg* gene in the medium and high ammonia nitrogen groups during the mid-stage were significantly higher than those in the control group. Furthermore, the gene expression level in the low ammonia nitrogen group was significantly higher than that in the other three groups (*p* < 0.05). By the end of the experiment, except for the medium ammonia nitrogen group, the gene expression levels decreased significantly with increasing concentration, and significant differences were observed among the four groups. Over time, the expression levels of all four genes decreased significantly.

### 3.7. Effects of Different Levels of Ammonia Nitrogen on the AMPK Pathway of Chinese Perch

**Figure 6:**
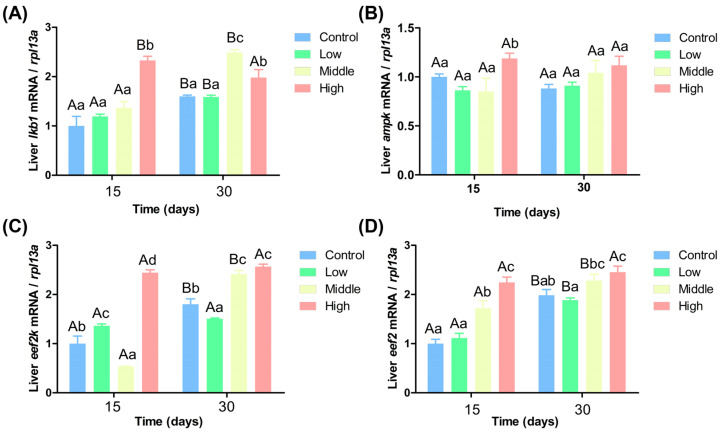
Expression of AMPK pathway-related genes under different ammonia nitrogen conditions. (**A**) Gene expression of *lkb1* in liver; (**B**) gene expression of *ampk* in liver; (**C**) gene expression of *eef2k* in liver; (**D**) gene expression of *eeef2* in liver. Significant differences in the same ammonia nitrogen group at different times were marked with different capital letters (*p* < 0.05), and significant differences among different ammonia nitrogen groups at the same time were marked with different lowercase letters (*p* < 0.05). Those marked with the same letter indicate no significant difference between groups (*p* < 0.05).

Ammonia nitrogen upregulated the expression of AMPK pathway-related genes in liver, with expression generally increasing with ammonia nitrogen concentration. Figure 6A shows that on the 15th day, *lkb1* gene expression in the high ammonia nitrogen group was significantly higher than in the other three groups (*p* < 0.05). On the 30th day, *lkb1* gene expression was significantly higher in the middle and high ammonia nitrogen groups than in the other two groups (*p* < 0.05). Over time, *lkb1* gene expression decreased significantly in the high ammonia nitrogen group but increased dramatically in the other three groups (*p* < 0.05). Figure 6B shows that on the 15th day, *ampk* gene expression in the high ammonia nitrogen group was significantly higher than in the other groups (*p* < 0.05). On the 30th day, there were no significant differences in *ampk* gene expression among groups, and no significant change over time (*p* > 0.05).

Figure 6C shows that on the 15th day, *eef2k* gene expression in the low and high ammonia nitrogen groups was significantly higher than in the other two groups, while its expression in the medium ammonia nitrogen group was significantly lower than in the control group (*p* < 0.05). On the 30th day, *eef2k* gene expression in middle and high ammonia nitrogen groups was significantly higher than in both the control and low concentration groups, while expression in the low ammonia nitrogen group was significantly lower than in the control group (*p* < 0.05). Over time, *eef2k* gene expression increased significantly in both the control and middle ammonia nitrogen groups (*p* < 0.05). Figure 6D indicates that on the 15th day, *eef2* gene expression in the middle and high ammonia nitrogen groups was significantly higher than in the other two groups (*p* < 0.05). On the 30th day, *eef2* gene expression in the high ammonia nitrogen group was significantly higher than in the control and low ammonia nitrogen groups (*p* < 0.05). Over time, *eef2* gene expression increased significantly in the control, low, and middle ammonia groups, but not in the high ammonia nitrogen group (*p* < 0.05).

## 4. Discussion

### 4.1. Experiment on the Acute Toxicity of Ammonia Nitrogen to Chinese Perch

The present study demonstrates a clear concentration-dependent relationship between ammonia nitrogen exposure and mortality in juvenile Chinese perch, with mortality rates escalating significantly from 0% to 100% as ammonia nitrogen concentrations increased from 10.39 to 15.03 mg/L over 96 h. These findings align with previous studies highlighting ammonia nitrogen as a critical environmental stressor in aquatic ecosystems, particularly for fish species with high sensitivity to nitrogenous wastes [8]. The calculated 96 h-LC_50_ value of 12.91 mg/L underscores the acute toxicity of ammonia nitrogen to juvenile Chinese perch, positioning this species within the range of sensitivity observed in other teleost fish. For instance, reported 96 h-LC_50_ values for ammonia nitrogen in freshwater fish such as *Cyprinus carpio* and *Oncorhynchus mykiss* typically range between 10 and 20 mg/L, depending on environmental conditions and life stages [19]. The significant mortality differences (*p* < 0.05) across concentration groups further validate the dose–response relationship, reinforcing ammonia nitrogen’s role as a potent toxicant. The observed mortality trends may be attributed to ammonia nitrogen’s multifaceted physiological impacts, including disruptions to ionoregulation, oxidative stress, and damage to gill epithelia, which impair respiratory and osmoregulatory functions [20]. At elevated concentrations, non-ionic ammonia (NH_3_), the toxic form of ammonia nitrogen, passively diffuses across gill membranes, leading to cellular acidosis and neurotoxicity [21]. Notably, the derived safe concentration (1.29 mg/L total ammonia nitrogen; 0.097 mg/L NH_3_) aligns with recommendations for aquaculture systems, where non-ionic ammonia levels below 0.1 mg/L are generally considered safe for chronic exposure [22]. In conclusion, these findings contribute to the growing body of evidence on ammonia nitrogen toxicity in aquatic organisms and provide actionable data for optimizing aquaculture protocols. By integrating LC_50_ values and safety thresholds into management practices, stakeholders can enhance the resilience of Chinese perch populations to ammonia nitrogen stress, promoting both ecological and economic sustainability.

### 4.2. Different Ammonia Nitrogen Conditions on the Chinese Perch Resisting Oxidative Stress and the Effects of Liver Enzyme Activity

**Figure 7:**
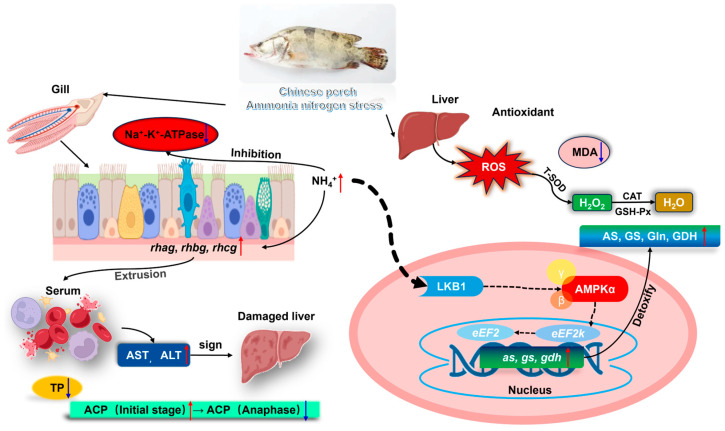
Effects of ammonia nitrogen stress on ammonia excretion and transformation of Chinese perch. Red arrows indicate an increase in enzyme activity and the expression levels of related genes in the pathway. Blue arrows represent a decrease in enzyme activity and the expression levels of related genes in the pathway. All the abbreviations in the figure can be found in the abbreviated list at the end of the text.

Fish respond to ammonia nitrogen stress by increasing oxidative stress production [15]. Elevated levels of ammonia nitrogen in aquaculture water can result in the generation of ROS in aquatic organisms [23]. ROS interact with membrane unsaturated fatty acids and cholesterol, leading to lipid peroxidation. This process reduces the fluidity of the phospholipid bilayer, ultimately causing an increase in cell membrane permeability [24,25]. Antioxidant enzymes are predominantly present in liver cells, with SOD acting as the primary defense mechanism against oxidative stress [26,27]. CAT further reduces H_2_O_2_ to H_2_O, minimizing its harmful effects. GSH aids in breaking down H_2_O_2_ [28], a byproduct of SOD activity, through the catalysis of GSH-Px and GST [29,30]. MDA is a product of free radical attack on membrane unsaturated fatty acids, which can lead to cell damage by interacting with proteins [31]. Under normal conditions, MDA content levels are low and can indicate the extent of free radical damage to cells. In this study, the levels of T-SOD, CAT, and GSH-Px activities, and MDA content generally increased with higher concentrations of ammonia nitrogen but decreased over time (Figure 7). Considering that the changes in pH values in water environment in each ammonia nitrogen group were not significant, the influence of pH on enzyme activity could be ignored. The conclusion is supported by research from Sun et al. [17]. These studies suggest that the activity of fish antioxidant enzymes tends to increase initially under stress and then decrease. However, Guan et al. observed the opposite trend in their study on the second-generation Chinese sturgeon (*Aclpenser Sinensis*), where they found that the antioxidant enzyme activity decreased with increasing concentrations of ammonia nitrogen [32]. Previous research has indicated that antioxidant enzyme activities are stimulated at low pollutant concentrations but are inhibited at high concentrations [33,34]. The discrepancy in findings may be attributed to the concentration levels within the tolerance range of Chinese perch in this study. When ammonia nitrogen levels exceed the fish’s tolerance threshold, the body reduces protein and amino acid decomposition to minimize NH_3_ accumulation, consequently lowering antioxidant enzyme activity [35]. In the current study, the liver antioxidant enzyme activity in Chinese perch showed a positive correlation with ammonia nitrogen concentration, primarily due to the fish’s response to low ammonia nitrogen conditions. Furthermore, the experiment revealed a gradual decrease in Chinese perch liver antioxidant enzyme activity over time, possibly due to prolonged exposure to stress, lead to ROS levels surpassing the antioxidant regulation threshold [36].

### 4.3. Different Ammonia Nitrogen Conditions Affect Chinese Perch Blood Physiological Indexes

Blood biochemical parameters are crucial indicators that reflect the physiological condition and overall health status of fish, serving as a fundamental tool for disease detection [37,38]. Chronic exposure to ammonia nitrogen stress leads to the infiltration of non-ionic ammonia from water into the bloodstream through the gills, subsequently impacting the enzymes and blood cells present in the serum [39]. In this experiment, there was no significant difference in the pH value of the water environment caused by the difference in ammonia nitrogen concentration, so the effect of pH value on enzyme activity in serum can be ignored. The plasma enzyme components include ACP and alkaline phosphatase (ALP), which are considered reliable and sensitive biological markers for assessing environmental stress-induced damage to fish liver and other organs. AST and ALT are crucial in indicating hepatopancreatic function and injury, serving as sensitive indicators of hepatocyte integrity [40]. Studies by Zhao et al. and Hoseini et al. observed significant increases in AST and ALT activities levels in fish exposed to ammonia nitrogen, suggesting potential cell membrane and liver damage [41,42]. Additionally, Zhang et al. reported a notable rise in ALP activity levels in *Megalobrama amblycephala* following exposure to ammonia nitrogen [43]. TP content is recognized as a dependable indicator of fish immune status and overall health [44]. Research by David et al. and Asthana et al. highlighted the decline in protein content in fish exposed to toxic substances, attributing it to cellular dysfunction or breakdown [45,46]. In an experimental setting, gene expression levels of AST, ALT and ALP activities in blood and liver increased with rising ammonia nitrogen concentrations, while TP levels decreased (Figure 7). Significant differences were observed among various groups in the later stages of the experiment. These findings align with previous studies by Li et al. and Tang et al., who investigated the impact of chronic ammonia nitrogen stress on blood parameters in *Pelteobagrus fulvidraco* [38,47]. In the experiment, it was observed that after 30 days of breeding, the high ammonia nitrogen content in the blood of ACP activity was significantly lower compared to the other three groups [38,48]. This occurrence can be attributed to the toxic excitatory effect generated in the fish’s body under the influence of ammonia nitrogen stress, leading to an increase in acid phosphatase synthesis in the blood. Consequently, the electrolyte balance in the blood is disrupted under high ammonia nitrogen stress, inhibiting the induction of acid phosphatase and resulting in a notable decrease in its activity. This finding aligns with previous research conducted by Jee et al., which also demonstrated a similar response in Chinese perch, indicating a certain level of tolerance to ammonia nitrogen in acid phosphatase activity, albeit significantly inhibited beyond a certain threshold [49]. Furthermore, the study revealed an increase in the levels of AST and ALT activities in the blood over time, while TP content levels decreased with time, showing significant variations among the different groups. In similar trends, ALT activity was observed in *Cyprinus carpio* under ammonia nitrogen stress [50]. This can be attributed to the accumulation of lipid peroxides in the fish body due to prolonged exposure to ammonia nitrogen, leading to cellular damage in the liver and pancreas, including cell separation from the basement membrane, cell wall rupture, cell swelling, and necrosis, ultimately impacting the normal physiological functions of these organs [51].

### 4.4. Impact of Varying Ammonia Nitrogen Conditions on Enzyme Activity and Gene Expression in Chinese Perch

Fish possess the ability to synthesize Gln through the enzymatic actions of GS and GDH, facilitating the conversion and transport of ammonia nitrogen, thereby mitigating ammonia nitrogen toxicity [52]. Additionally, fish can transform ammonia into less toxic compounds through the active excretion of ammonium ions [21]. The gill epithelium is rich in chloride cells that play a crucial role in the excretion of ammonium ions. The basolateral membrane of these cells exhibits elevated levels of NH_4_^+^-sensitive Na^+^-K^+^-ATPase activity and Na^+^-K^+^-2Cl^−^ cotransporters, both of which are instrumental in the intracellular transfer of ammonium ions [53]. In the present study, the activity of Na^+^-K^+^-ATPase in the three experimental groups was significantly lower than that observed in the control group, while the expression levels of the *rhag*, *rhbg*, and *rhcg* genes were markedly elevated compared to the control group. Notably, the expression of *rhag* and *rhbg* increased in correlation with rising ammonia nitrogen concentrations (Figure 7). This finding aligns with the research conducted by Hung et al., which reported a significant upregulation of *rhbg* gene expression in the muscle and liver of the mangrove *Kryptolebias marmoratus* following ammonia nitrogen exposure [54]. Similarly, a significant increase in the expression of *rhbg1* and *rhcg1* in the gills of climbing perch subjected to ammonia nitrogen stress [55]. Furthermore, *Oncorhynchus mykiss* exhibited a notable increase in *rhcg2* expression within gill tissues under ammonia nitrogen stress conditions [52]. Moreover, the expression levels of Na^+^-K^+^-ATPase activity, *rhag*, *rhbg*, and *rhcg* exhibited a significant decline over time, corroborating findings from previous studies [56].

### 4.5. Effects of Different Ammonia-Nitrogen Conditions on Ammonia Excretion and Transformation in Chinese Perch

Fish exhibit various mechanisms to assimilate free ammonia, including preventing its accumulation within the body, reducing its production, increasing its excretion, or converting it into less toxic compounds [57]. Gln serves as a precursor and plays a crucial role in cellular metabolism [58,59,60,61,62]. GDH and GS are key enzymes in the Gln synthesis pathway [63] and represent important ammonia nitrogen detoxification strategies in fish [64,65]. Numerous studies have reported that fish respond to ammonia nitrogen exposure with increased brain GS activity and Gln levels [66,67]. In the present study, the activities of AS, GS, Gln, and GDH in the liver increased with the rise in water ammonia-nitrogen concentration. The expression levels of the *as, gs*, and *gdh* genes in the liver were consistent with the enzyme activities and also increased with ammonia nitrogen concentration (Figure 7). For example, in *Monopterus albus*, Gln levels in the liver and muscle, as well as GS activity in the gut and liver, significantly increased after 144 h of high ammonia nitrogen exposure [68]. In *Bostrychus sinensis*, muscle Gln content significantly increased after 144 h of ammonia nitrogen exposure [69]. In *Oplopanax elatus Nakai*, Gln content increased significantly with GS activity after ammonia nitrogen stress [70]. In *Litopenaeus vannamei*, GDH and GS activities in the gill tissues and GDH activity in the hepatopancreas significantly increased after acute ammonia nitrogen stress [71]. These findings are consistent with the results of the present study. In response to ammonia nitrogen exposure, Chinese perch can also detoxify by generating Gln. Over time, the enzyme activities of AS, GS, and GDH in the liver generally decreased, and there was no significant difference among the four groups. Except for the high ammonia nitrogen group, the expression levels of the *as* and *gs* genes in the liver decreased over time, while the expression of the *gdh* gene increased.

### 4.6. Effects of Different Ammonia Nitrogen Conditions on the AMPK Pathway in Chinese Perch

The AMP-activated protein kinase (AMPK) gene plays a crucial regulatory role in energy metabolism within organisms. Low-temperature stress has been shown to upregulate the expression of AMPK in *Cherax quadricarinatus* [72]. In this study, ammonia nitrogen activated the AMPK pathway by upregulating the *lkb1* gene. In the liver, the expression levels of *lkb1, ampk*, *eef2k*, and *eef2* showed a positive feedback trend with ammonia nitrogen concentration. Over time, there was no significant difference in the content of *ampk* in the liver, while the expression levels of *lkb1*, *eef2k* and *eef2* generally increased (Figure 7). When fish are exposed to oxidative stress, energy and substances are used for growth, and the growth rate of fish decreases [73]. In this study, ammonia nitrogen exposure increased the activity of antioxidant enzymes in fish, diverting energy and substances towards oxidative stress responses. This resulted in energy deficiency in the organism, thereby upregulating the AMPK-related pathway and increasing the expression levels of *lkb1, ampk, eef2k*, and *eef2*. This conclusion is consistent with the fact that Chinese perch exposed to DO (3 mg/L) for a long time produced oxidative stress, resulting in faster ammonia nitrogen transport, impaired tricarboxylic acid cycle and insufficient energy, and increased expression of AMPK-related pathway genes [7].

### 4.7. Outlook

This study focuses on the effects of ammonia nitrogen concentration on the enzyme activity and gene expression of Chinese perch, with particular emphasis on the toxicological impacts on the gills, liver, and blood. However, the influence of ammonia nitrogen on the intestinal system is equally significant. Future research will prioritize the examination of intestinal tissue morphology, intestinal microbiota, and intestinal physiology in relation to ammonia nitrogen exposure. Furthermore, it is important to note that ammonia nitrogen in aquatic environments can be converted into other compounds, such as nitrites and nitrates, through microbial processes. Therefore, the effects of ammonia nitrogen on the composition and diversity of aquatic microorganisms warrant further exploration.

## 5. Conclusions

This study systematically investigated the acute toxicity and physiological responses of juvenile Chinese perch under ammonia nitrogen stress. The 96 h-LC_50_ of ammonia nitrogen was determined as 12.91 mg/L (non-ionic ammonia: 0.097 mg/L), with a safe concentration of 1.29 mg/L, highlighting its lower tolerance compared to other aquaculture species, necessitating stringent water quality management in farming practices. Ammonia nitrogen exposure induced oxidative stress, evidenced by dose-dependent increases in hepatic antioxidant enzymes (T-SOD, CAT, and GSH-Px) and lipid peroxidation (MDA), though prolonged exposure led to enzymatic inhibition, indicating cumulative oxidative damage. Serum biochemical markers (AST, ALT, and ACP) rose significantly, while total protein declined, reflecting hepatopancreatic dysfunction. Enhanced ammonia nitrogen detoxification was achieved via upregulated hepatic enzyme activities (AS, GS, and GDH) and glutamine synthesis, supported by elevated expression of ammonia nitrogen metabolism-related genes (*as*, *gs*, and *gdh*). Gill ammonia excretion mechanisms were modulated through suppressed Na^+^-K^+^-ATPase activity but increased expression of ammonia transporters (*rhag*, *rhbg*, and *rhcg*). Activation of the AMPK pathway (*lkb1*, *ampk*, *eef2k*, and *eef2*) underscored adaptive energy reallocation under stress. These findings provide critical insights into ammonia nitrogen toxicity mechanisms in Chinese perch and establish a foundation for optimizing aquaculture protocols to mitigate ammonia nitrogen related risks. Future studies should explore interorgan metabolic coordination and molecular networks governing ammonia resilience in this species.

## Figures and Tables

**Table 1:** Real-time primer sequence for Chinese perch.

Gene	Primer	Primer Sequence (5′-3′)	Tm (°C)
*gdh*	F	CATTTCCTTCCCGTGTT	58
	R	TCTGTCTGCGGAGTTGGT	
*ampd*	F	CATTTCCTTCCCGTGTT	58
	R	TCTGTCTGCGGAGTTGGT	
*ast*	F	TCTGTGCTCAGTCGTTCTC	58
	R	AACTCGCTTCAGGTTGTCT	
*alt*	F	ATGTCCGAGAATGGAGTGT	58
	R	TCAGGGTAGGAGCAGAGC	
*gs*	F	TGGATTGATGGAACTGGAGAG	58
	R	CCACTCAGGCAGGTCTTC	
*as*	F	TGCTGCTACACTGGTGAAG	58
	R	GCGATGATGTCTGGACTGT	
*ampk*	F	GGGATGCAAACCAAGATG	58
	R	ACAGACCCAGAGCGGAGA	
*eef2*	F	TCTGCTGTTATCCCGCCT	58
	R	TCGCCATCACTCCTCCTCT	
*lkb1*	F	GACGGGGCACTTAAAATC	58
	R	GTGTTACTCCAGCAGACCAAA	
*rpl13a*	F	CACCCTATGACAAGAGGAAGC	59
	R	TGTGCCAGACGCCCAAG	
*rhag*	F	TGATTGGATTAGTGGCTGGCATA	58
	R	GTGGACACCGCAGGTATCTT	
*rhbg*	F	AAGACGCAGCAACCAACAT	58
	R	CCAAGGCACCGAAGAGGAT	
*rhcg*	F	ACATCCAGAACTCCACTCTT	60
	R	AGATGACACCACAGCAGAA	

Note: Tm: Melting temperature.

**Table 2:** Effect of different ammonia nitrogen concentrations on the mortality of juvenile Chinese perch.

Ammonia Nitrogen Concentration (mg/L)	Mortality (%)
10.39 ± 0.40	0 ^a^
11.84 ± 0.43	20 ± 0 ^b^
13.46 ± 0.54	60 ± 5.77 ^c^
15.03 ± 0.42	100 ± 0 ^d^

Note: Different lowercase superscripts indicate significant differences (*p* < 0.05) among treatments.

## Data Availability

All data are available from the corresponding author by request.

## Abbreviations

The following abbreviations are used in this manuscript:

ACP	Acid phosphatase
ALT	Alanine aminotransferase
AMPK	AMP-activated protein kinase
AS	Asparagine synthase
AST	Aspartate aminotransferase
CAT	Catalase
eEF2	Eukaryotic elongation factor 2
eEF2k	Eukaryotic elongation factor 2 Kinase
GDH	Glutamate dehydrogenase
Gln	Glutamine
GS	Glutamine synthetase
GSH-Px	Glutathione peroxidase
LKB1	Liver kinase B1
MDA	Malondialdehyde
Rhag	Rh a glycoprotein
Rhbg	Rh b glycoprotein
Rhcg	Rh c glycoprotein
TP	Total Protein
T-SOD	Total Superoxide Dismutase
